# Superiority of high sensitivity cardiac troponin I over NT-proBNP and adiponectin for 7-year mortality in stable patients receiving haemodialysis

**DOI:** 10.1038/s41598-024-62491-4

**Published:** 2024-05-20

**Authors:** Nanami Iwamura, Shuhei Kidoguchi, Nanae Asahi, Izumi Takeda, Kohei Matsuta, Kyoko Miyagi, Masayuki Iwano, Ryoichi Miyazaki, Hideki Kimura

**Affiliations:** 1 Department of Clinical Laboratory, University of Fukui Hospital, 23-3 Matsuoka-Shimoaizuki, Eiheiji, Yoshida, Fukui, 910-1193 Japan; 2 Department of Internal Medicine, Fujita Memorial Hospital, Fukui, Japan; 3 Division of Nephrology, Department of General Medicine, School of Medicine, University of Fukui, Fukui, Japan

**Keywords:** Biomarkers, Nephrology

## Abstract

Patients on haemodialysis (HD) have high mortality risk, and prognostic values of the major cardiovascular biomarkers cardiac troponin I (cTnI), N-terminal pro-brain natriuretic peptide (NT-proBNP), and adiponectin should be ascertained over longer follow-up periods using higher-sensitivity assays, which we undertook. In 221 HD patients, levels of high-sensitivity (hs)-cTnI, NT-proBNP, and adiponectin, were measured using high-sensitivity assays, and their associations with all-cause mortality (ACM) and cardiovascular mortality (CVM) were prospectively investigated for 7 years. Higher hs-cTnI and NT-proBNP levels were significant risk factors for ACM and CVM in the Kaplan–Meier analysis. Multivariate Cox proportional hazards analyses in a model including hs-cTnI and NT-proBNP identified log hs-cTnI, but not log NT-proBNP, as an independent risk factor for ACM (HR 2.12, *P* < 0.02) and CVM (HR 4.48, *P* < 0.0005). Stepwise analyses identified a high hs-cTnI tertile as a risk factor for ACM (HR 2.31, *P* < 0.01) and CVM (HR 6.70, *P* < 0.001). The addition of hs-cTnI to a model including age, CRP, DM, and NT-proBNP significantly improved the discrimination of ACM and CVM each over 7 years. Conclusively, hs-cTnI was superior to NT-proBNP and adiponectin in predicting ACM and CVM over 7 years in HD patients, suggesting the significance of baseline hs-cTnI measurements in long-term management.

## Introduction

Compared to the general population, patients who undergo haemodialysis (HD) have a poorer prognosis, with higher rates of cardiovascular morbidity and mortality^1^. The identification of clinical factors with high prognostic value for long-term all-cause and cardiovascular mortality rates may provide a useful tool for individualised management and better prognosis of patients who undergo dialysis. Biomarkers of cardiovascular diseases are associated with poor prognosis in patients on HD, and the association between several biomarkers and prognosis has been previously analysed^2,3^. Recent studies have focused on cardiac troponin associated with cardiac infarction or ischaemia and the N-terminal pro-brain natriuretic peptide (NT-proBNP) levels, which reflect left ventricular overload^4–6^. Cardiac troponins (cTn) include type I troponins (cTnI), and type T troponins (cTnT) that are released into the blood during the early phase of myocardial infarction and serve as injury biomarkers^7^. cTnI inhibits actin–myosin interaction, whereas cTnT exists on tropomyosin and mediates tropomyosin–troponin binding^7^. Compared with cTnT, cTnI is less affected by renal clearance and better reflects the cardiac pathology in patients on dialysis^8^; increased cTnI levels are associated with all-cause and cardiovascular mortality^4,9^. In contrast, NT-proBNP, which is secreted by cardiomyocytes in response to left ventricular wall stress^2,3,9^ and predominantly undergoes renal clearance, is used to diagnose heart failure and evaluate prognosis in the general population^10,11^. In patients on dialysis, NT-proBNP has prognostic value for predicting all-cause and cardiovascular mortality^6,12^.

Adiponectin is an adipocytokine that mediates anti-atherosclerotic effects in the general population; however, in patients who have renal failure, need dialysis, and are susceptible to atherosclerotic complications, adiponectin blood levels are elevated due to decreased renal clearance. Though high adiponectin levels are an independent prognostic risk factor for mortality in renal failure^5,13^, their positive correlation with NT-proBNP attenuates the prognostic power when adjusted for NT-proBNP^13^.

Furthermore, in HD patients, cTnI and NT-proBNP are potent prognostic risk factors, and adiponectin is paradoxically associated with poor prognosis. Few studies have simultaneously analysed the prognostic value of cTnI, NT-proBNP, and adiponectin in patients undergoing HD, and no study has measured cTnI accurately with a measurement sensitivity of several pg/mL and, determined high-sensitivity (hs)-cTnI levels, and evaluated their long-term predictive ability for mortality in comparison with other biomarkers. Therefore, a close evaluation of the prognostic value of these three biomarkers for potential application in routine tests could be beneficial for the practical management of patients undergoing dialysis.

In this study, we aimed to accurately quantify the baseline blood levels of hs-cTnI, NT-proBNP, and adiponectin in clinically stable patients on maintenance HD and prospectively analyse their prognostic value for all-cause and cardiovascular mortality during a 7-year follow-up period.

## Methods

This prospective observational cohort study included 221 patients receiving maintenance HD at the dialysis centre of Fujita Memorial Hospital who were enrolled in March 2014 based on their not fulfilling the following exclusion criteria: age < 18 years; dialysis vintage < 3 months; acute-phase heart failure; history of pulmonary embolism; acute myocardial infarction; major surgery within the past 3 months; acute infections within the last month, and CRP ≥ 1 mg/dL. Regarding the occurrence of all-cause and cardiovascular mortality, the participants were followed up for a median duration of 7.0 years, from March 2014 to February 2021. In this cohort, HD had been initiated because of end-stage renal disease due to chronic glomerulonephritis (n = 85), diabetic nephropathy (n = 65), nephrosclerosis (n = 22), polycystic kidney disease (n = 16), chronic pyelonephritis (n = 4), rapidly progressive glomerulonephritis (n = 5), lupus nephritis (n = 3), pre-eclampsia (n = 2), other aetiology (n = 8), and idiopathic causes (n = 11). The participants underwent a 3- to 5-h HD session thrice-weekly, generally using high-flux membranes and standard heparin doses. By modulating blood flow rates (QB) and BP-lowering drugs, the BP levels of individual patients during dialysis were adjusted to be above 100 mmHg and to achieve a Kt/V index of 1.2 or more. The QB was generally set at 200 to 250 mL/min. Smoking was defined as current or habitual cigarette smoking. Hypertension was defined as a systolic blood pressure > 140 mmHg, diastolic pressure > 90 mmHg, or the use of antihypertensive drugs.

### Ethics declaration

The ethics committees of Fujita Memorial Hospital (Approval No. 24) and the University of Fukui Hospital (Approval No. 20120137) approved the study protocol. Written informed consent was obtained from all participants. All procedures followed the Declaration of Helsinki and the ethical standards of the Committee on Human Experimentation.

### Clinical data and laboratory measurements

Clinical data were acquired at the time of study entry, and included age, sex, cardiovascular risk factors, and comorbidity (history of cardiovascular diseases, diabetes mellitus, and/or hypertension); blood chemistry; nutritional data, including the Geriatric Nutritional Risk Index (GNRI)^14^ and normalized protein catabolic rate (nPCR)^15^; and HD data, including vintage, session time and frequency of dialysis, Kt/V^15^, and the Erythropoietin Resistance Index [ERI = a 4-week total dose of erythropoietin (μg) / DW(kg) / Hb (g/dL)]. Estimated glomerular filtration rate (eGFR) values [eGFR (mL/min/1.73 m^2^) = 194 × Serum Cr^−1.094^ × Age^−0.287^ × 0.739 (if female)] were calculated from the Japanese equation^16^ and considered a marker of residual renal function. Venous blood samples were collected from all participants immediately before dialysis after a long interdialytic interval (single collection). Serum levels of Cr, total cholesterol (TC) and triglycerides (TG) were measured using a standard enzymatic method. Serum levels of low density lipoprotein cholesterol (LDL-C) and high density lipoprotein cholesterol (HDL-C) were measured by direct and chemical methods using commercial kits (Cholest-LDL and Cholestest-N HDL, respectively; Sekisui Medical, Tokyo, Japan). Serum albumin levels were determined using a bromocresol green dye-binding assay. hs-cTnI values were evaluated using the ARCHITECT i2000SR Diagnostic System (Abbott STAT High Sensitive Troponin I assay, Abbott Diagnostics, USA); the limit of detection (LOD) was 1.1 ng/L, and the limit of quantification (LOQ), with coefficients of variation of less than 10%, was 2.7 ng/L. The cut-off value at the 99th percentile of the reference population was 26.2 ng/L^17^. NT-proBNP levels were measured using a Cobas 8000 analyser (NT-proBNP II, Roche Diagnostics K.K., Swiss Confederation); the LOD and LOQ were 5 and 50 pg/mL, respectively. Total adiponectin levels were determined using a novel developed automated homogenous assay (Denka Seiken Co., Ltd., Tokyo, Japan) on a TBA-c16000 chemistry analyser (Canon Medical Systems Corp., Japan); the LOD and LOQ were 0.164 and 0.263 μg/mL, respectively.

### Statistical analysis

Continuous variables are expressed as the mean and standard deviation (SD) when normally distributed or as the median and interquartile range when not normally distributed. Relationships between clinical variables were examined using Pearson's correlation analysis and univariate and multivariate linear regression analyses. Multivariate linear regression analysis, with and without forward stepwise variable selection, was performed to determine the independent predictors of hs-cTnI, NT-proBNP, and adiponectin levels. Patients were stratified into tertiles (low, middle, and high) according to their baseline hs-cTnI, NT-proBNP, and adiponectin levels. The intergroup differences in continuous variables and among the three groups were assessed using the unpaired *t*-test or Mann–Whitney *U* test and ANOVA with multiple comparisons, respectively. The chi-square test was used to assess intergroup differences in categorical variables. The trend of clinical factor values among 3-tertile groups was analyzed by linear regression analysis using averages (trend test) for continuous variables and the Cochran–Armitage trend test for categorical variables. A Kaplan–Meier time-to-event curve with a log-rank test was used to compare outcomes (all-cause or cardiovascular mortality) among the tertiles of hs-cTnI, NT-proBNP, and adiponectin. To identify variables that were independently associated with mortality, univariate and multivariable proportional hazards [hazard ratios (HR) and 95% confidence intervals (CI)] were calculated using Cox proportional regression analysis, with and without forward stepwise variable selection. The C-indices of hs-cTnI and NT-proBNP for prognostic performance were calculated for every year of follow-up (Years 1–7). Cut-off values were calculated by maximising the sensitivity and specificity. Furthermore, the Net Reclassification Index (NRI) and integrated discrimination improvement (IDI) were estimated to examine whether the addition of hs-cTnI or NT-proBNP to the main model with a corresponding alternative biomarker could significantly improve the reclassification and discrimination of patients. Statistical significance was defined by *P* < 0.05 (two-tailed tests). The statistical software packages SPSS version 24 (IBM Corp., Armonk, NY, USA) and R Package ver. 4.3.1 (www.R-project.org) were used for statistical analysis.

## Results

### Baseline characteristics of the cohort

A total of 221 clinically stable adult patients (age ≥ 18 years) who were 138 men and received maintenance HD for 3 months or longer were enrolled in this study. The median follow-up duration was 7.0 years. The average age of the participants was 66.8 years, and 138 (62%) were men. Sixty-five patients (29.4%) had diabetes mellitus (DM), and 73 patients (33%) had a history of cardiovascular diseases, including myocardial infarction (18%). The baseline clinical characteristics of the whole cohort are summarized in Table 1.

**Table 1:** Baseline characterisitics of the study cohort and tertiles of hs-cTnI and NT-proBNP.

	Total (n = 221)	Tertiles of hs-cTnI^a^				Tertiles of NT-proBNP^b^			
		Low (n = 73)	Middle (n = 74)	High (n = 74)	*P-*value^c^	Low (n = 73)	Middle (n = 74)	High (n = 74)	*P*-value^c^
Age, years	66.8 ± 12.5	62.5 ± 11.6	68.2 ± 11.9	69.8 ± 12.8	< 0.001	62.2 ± 12.8	68.7 ± 11.0	69.9 ± 12.3	< 0.001
Sex (men/women)	138/83	43/30	52/22	43/31	0.915	49/24	47/27	42/32	0.194
HD vintage, months	119.0 ± 107.2	116.4 ± 107.9	108.6 ± 101.7	131.9 ± 11.9	0.382	110.0 ± 101.8	118.6 ± 105.6	128.2 ± 114.3	0.305
DM, n (%)	65 (29.4%)	15 (20.5%)	23 (31.1%)	27 (36.5%)	0.034	13 (17.8%)	21 (28.4%)	31 (41.9%)	0.001
Hypertension, n (%)	196 (88.7%)	64 (87.7%)	68 (91.9%)	64 (86.5%)	0.817	64 (87.7%)	70 (94.6%)	62 (83.8%)	0.452
Smoking status, n (%)	37 (16.7%)	13 (17.8%)	12 (16.2%)	12 (16.2%)	0.796	13 (17.8%)	13 (17.6%)	11 (14.9%)	0.632
Anti-lipidemic drugs, n (%)	37 (16.7%)	11 (15.1%)	10 (13.5%)	16 (21.6%)	0.286	14 (19.2%)	10 (13.5%)	13 (17.6%)	0.796
History of CVD, n (%)	73 (33.0%)	16 (21.9%)	21 (28.4%)	36 (48.6%)	< 0.001	15 (20.5%)	28 (37.8%)	30 (40.5%)	0.010
History of MI, n (%)	40 (18.1%)	7 (9.6%)	12 (16.2%)	21 (28.4%)	0.003	8 (11.0%)	14 (18.9%)	18 (24.3%)	0.035
BMI, kg/m^2^	21.8 ± 3.9	21.4 ± 3.2	22.0 ± 3.6	22.0 ± 4.7	0.384	22.9 ± 4.1	21.2 ± 3.2	21.4 ± 4.1	0.016
GNRI	93.9 ± 7.0	95.2 ± 5.5	93.9 ± 7.1	92.6 ± 8.0	0.028	96.2 ± 6.2	93.0 ± 6.7	92.5 ± 7.4	0.001
ERI, μg/Hb (g/dL)/DW (kg)	0.25 ± 0.17	0.22 ± 0.16	0.25 ± 0.19	0.28 ± 0.15	0.019	0.19 ± 0.15	0.26 ± 0.16	0.30 ± 0.17	< 0.001
CRP, mg/dL^d^	0.07 (0.03–0.21)	0.05 (0.03–0.10)	0.08 (0.03–0.22)	0.10 (0.05–0.37)	< 0.001	0.06 (0.03–0.16)	0.06 (0.03–0.13)	0.12 (0.05–0.37)	< 0.001
Ca, mg/dL	8.7 ± 0.7	8.7 ± 0.6	8.8 ± 0.7	8.7 ± 0.7	0.893	8.7 ± 0.6	8.7 ± 0.7	8.7 ± 0.7	0.660
iP, mg/dL	5.6 ± 1.4	5.4 ± 1.3	5.7 ± 1.4	5.6 ± 1.6	0.270	5.6 ± 1.3	5.7 ± 1.5	5.4 ± 1.5	0.408
eGFR, mL/min/1.73 m^2e^	3.92 (3.42–4.92)	3.70 (3.28–4.42)	3.91(3.44–5.07)	4.22 (3.59–5.38)	0.020	3.64 (3.22–4.65)	3.87 (3.33–4.86)	4.24 (3.67–5.40)	0.017
Kt/V	1.32 ± 0.33	1.32 ± 0.34	1.30 ± 0.37	1.35 ± 0.29	0.604	1.27 ± 0.32	1.33 ± 0.37	1.37 ± 0.31	0.075
PCR, g/kg	50.5 ± 15.3	49.8 ± 15.3	50.5 ± 13.2	51.2 ± 17.2	0.581	54.0 ± 15.0	49.3 ± 14.7	48.3 ± 15.7	0.025
TG, mg/dL^d^	91.5 (65.4–134.6)	99.9 (72.2–143.6)	92.5 (69.8–122.7)	82.3 (63.2–116.7)	0.165	93.8 (65.4–151.5)	90.5 (68.0–125.4)	83.7 (64.7–119.6)	0.159
HDL, mg/dL	44.1 ± 12.5	44.9 ± 14.7	43.2 ± 11.2	44.1 ± 11.5	0.694	0.5	45.4 ± 12.2	43.0 ± 10.1	0.734
LDL, mg/dL	83.6 ± 26.1	83.8 ± 23.9	84.7 ± 27.1	82.4 ± 27.4	0.748	85.2 ± 23.9	83.5 ± 27.1	82.2 ± 27.5	0.492
Adiponectin mg/mL	20.0 ± 10.8	16.7 ± 7.8	20.0 ± 11.0	23.2 ± 12.2	< 0.001	16.1 ± 8.0	21.0 ± 11.2	22.8 ± 11.7	< 0.001
hs-cTnI ng/L^d^	19.2 (12.1–36.8)	9.2 (7.1–12.0)	19.2 (16.6–24.0)	49.6 (36.9–75.7)	< 0.001	12.2 (7.5–20.6)	17.1 (12.5–28.8)	40.9 (24.8–67.6)	< 0.001
Men	20.2 (12.1–34.9)	10.0 (7.4–12.0)	20.2 (17.2–24.2)	50.1 (37.3–75.1)	< 0.001	13.2 (8.0–23.5)	17.3 (12.2–24.3)	48.5 (26.0–71.2)	< 0.001
Women	17.6 (11.9–40.0)	8.7 (6.7–12.4)	17.4 (15.9–23.3)	46.8 (36.9–76.9)	< 0.001	10.0 (7.3–15.2)	16.0 (13.2–31.1)	38.7 (19.0–63.4)	< 0.001
NT-ProBNP pg/mL^d^	4370.0 (1890.0–9520.0)	2260.0 (1070.0–4140.0)	3480.0 (1962.5–7180.0)	11,150.0 (5482.5–27,100.0)	< 0.001	1240.0 (825.0–1880.0)	4370.0 (3215.0–5410.0)	18,200.0 (9537.5–33,375.0)	< 0.001

Continuous variables are expressed as the mean ± SD or median (interquartile range) whereas categorial variables are expressed as the frequency (proportion).

*HD* haemodialysis, *DM* diabetes mellitus, *CVD* cardiovascular disease, *MI* myocardial infarction, *BMI* body mass index, *GNRI* Geriatric Nutritional Risk Index, *ERI* Erythropoietin Resistance Index, *CRP* C-reactive protein, *PCR* protein catabolic rate, *hs-TnI* high-sensitivity cardiac troponin I, *NT-proBNP* N-terminal pro-brain natriuretic peptide.

^a^The tertiles of hs-cTnI were 14.6 and 31.1 ng/L.

^b^The tertiles of NT-proBNP were 2570 and 7570 pg/mL.

^c^*P*-values indicate p-trend by linear regression for continuous variables and the Cochran-Armitage trend test for categorial variables.

^d^log10-transformed before analysis.

^e^Natural log-transformed before analysis.

Baseline pre-dialysis blood levels of clinical parameters, including biomarkers, were accurately measured. Hs-cTnI, NT-proBNP, and adiponectin measurements exceeded the corresponding LOQs in 99.5%, 100%, and 100% of participants, respectively, thereby ensuring the accuracy of the measured values. In the cohort of overall, men, and women, 84 (38%), 37 (26.8%), and 49 (59.0%) participants had higher hs-cTnI levels than the corresponding 99th percentile of the healthy population (26.2 ng/L for overall, 34.2 ng/L for men, and 15.6 ng/L for women), respectively^17,18^. Of all participants, 123 (55.6%) had higher NT-proBNP levels than the 99th percentile of 3592 pg/mL for patients with renal dysfunction (eGFR < 60 mL/min/1.73 m^2^)^19^. Regarding adiponectin, only one patient had a lower level than the cut-off value for metabolic syndrome (4 μg/mL)^20^, and 184 patients (83.2%) had higher levels than the upper reference range for the healthy population (10 μg/mL)^21^. These results are similar to those of previous reports^8,9,21–23^. The clinical characteristics at baseline of the sub-cohorts categorised by tertiles for hs-cTnI of and NT-proBNP levels are shown in Table 1.

### Correlations between biomarkers and other factors

Participants with higher hs-cTnI and NT-proBNP levels were significantly older and had significantly higher rates of DM, pre-existing cardiovascular diseases, and myocardial infarction; significantly higher levels of ERI, CRP, eGFR and adiponectin; and significantly lower levels of GNRI (Table 1). Patients with higher hs-cTnI levels had significantly higher NT-proBNP levels, whereas those with higher NT-proBNP levels had significantly higher hs-cTnI levels and significantly lower levels of body mass index (BMI) and PCR (Table 1). The median values of hs-cTnI were higher in men than in women overall and in each tertile of hs-cTnI and NT-proBNP, but the gender difference proved not to be statistically significant using the Mann–Whitney U test (Table 1).

The relationships between hs-cTnI, NT-proBNP, or adiponectin and the clinical parameters were evaluated using linear regression analyses (results in Supplementary Table S1A–C). Independent determinants for each biomarker were identified using compulsory and stepwise multiple linear regression analyses in a model that included factors with a significant univariate correlation. Independent determinants for hs-cTnI levels were the presence of cardiovascular diseases (standardised partial regression coefficient (β) = 0.145, P < 0.05) and NT-proBNP levels (β = 0.534, *P* < 0.001), those for NT-proBNP levels were the presence of DM (β = 0.195, *P* < 0.001), BMI (β =  − 0.269, *P* < 0.001), CRP levels (β = 0.151, *P* < 0.01), and hs-cTnI levels (β = 0.497, *P* < 0.001), and those for adiponectin levels were male sex (β =  − 0.237, *P* < 0.001), BMI (β =  − 0.229, *P* < 0.001), HDL-C levels (β = 0.285, *P* < 0.001), TG levels (β =  − 0.144, *P* < 0.05), and hs-cTnI levels (β = 0.223, *P* < 0.001). hs-cTnI levels were predominantly associated with NT-proBNP levels, whereas NT-proBNP levels were associated with several other parameters, including DM presence, BMI, CRP levels, and hs-cTnI levels. Adiponectin levels were positively associated with HDL-C levels and negatively associated with male sex, TG, and BMI levels; moreover, adiponectin was positively associated with hs-cTnI levels.

In univariate but not multivariate regression analyses, eGFR levels were slightly but significantly positively associated with levels of hs-cTnI (β = 0.155, P < 0.05) and NT-proBNP (β = 0.137, P < 0.05) (Tables S1A and S1B). eGFR levels were also associated positively with age (β = 0.288, P < 0.001) and CRP levels (β = 0.216, P < 0.005) and negatively with GNRI values (β = − 0.349, P < 0.001) in the study participants. Since higher age, higher CRP, and lower GNRI were related to higher hs-cTnI and NT-proBNP (Tables S1A,B), these associations may have caused a weak positive and paradoxical association of eGFR with hs-cTnI and NT-proBNP. The Cr-based eGFR values^16^ and serum Cr levels showed a strong inverse correlation. Considering that in HD patients, Cr generation is positively associated with good nutrition status (younger age and higher BMI and albumin levels)^24^, eGFR levels may be associated negatively with nutrition status, such as age and GNRI, and may only marginally reflect renal function in the participants, a large part of whom were considered anuria. Hence, eGFR levels were probably not an accurate marker of residual renal function and were not analyzed as candidate risk factors for clinical outcomes.

### Clinical outcomes based on baseline levels of each biomarker

**Figure 1:**
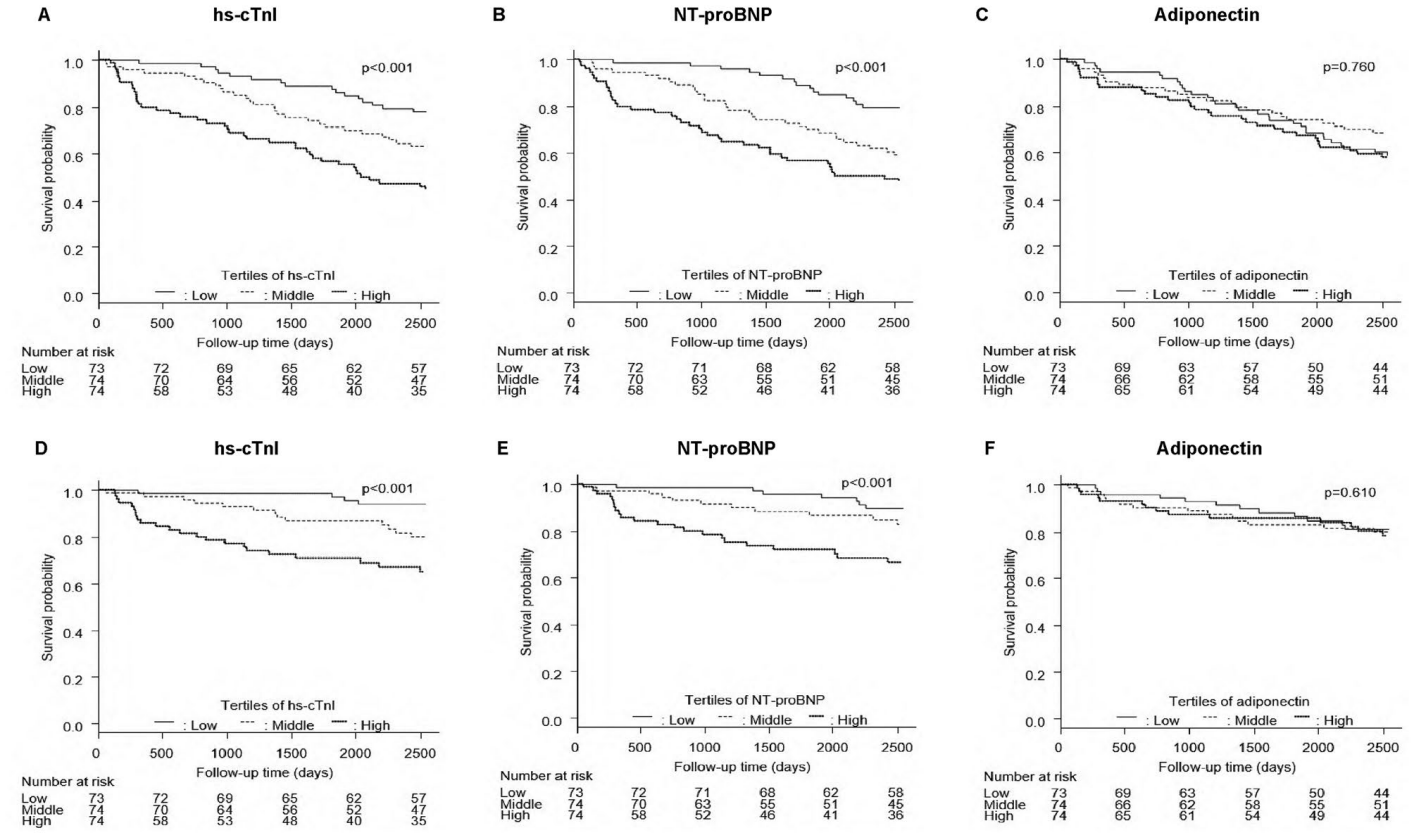
Kaplan–Meier survival curve analyses of all-cause mortality (ACM) and cardiovascular mortality (CVM) in patients stratified according to the tertiles of baseline values of hs-cTnI (**A**, **D**), NT-proBNP (**B**, **E**), and adiponectin (**C**, **F**). hs-cTnI, high-sensitivity troponin I; NT-proBNP, N-terminal pro-B-type natriuretic peptide. The tertiles of hs-cTnI, NT-proBNP, and adiponectin levels were 14.6 and 31.1 ng/L; 2570 and 7570 pg/mL; and 13.8 and 23.1 μg/mL, respectively.

During the median 7.0-year follow-up period of our study participants (n = 211), 84 participants (38.0%) died (Table S2A). Among these, 40 (47.6%) died of cardiovascular diseases (Table S2B). Kaplan–Meier analyses were performed to investigate the associations of the tertiles for each biomarker with the outcomes (Fig. 1). As shown in Fig. 1A and D, all-cause mortality (ACM) and cardiovascular mortality (CVM) rates were significantly higher in the middle (14.6–30.9 ng/L, *P* = 0.041 and *P* = 0.016, respectively) and high (31.1–1921.0 ng/L, *P* < 0.001 for both) tertiles of hs-cTnI compared with the low tertile of hs-cTnI (2.3–14.4 ng/L), and in the high tertile (*P* = 0.013 and *P* = 0.026, respectively) compared with the middle tertile. Regarding the tertiles of NT-proBNP (Fig. 1B,E), the ACM rates were significantly higher in the middle (2570–7260 pg/mL, *P* = 0.006) and high (7570–135,000 pg/mL, *P* < 0.001) tertiles than in the low tertile (103–2569 pg/mL), whereas CVM rates were significantly higher in the high (*P* < 0.001 and *P* = 0.017, respectively) tertile than in the low and middle tertiles. For adiponectin (Fig. 1C,F), no significant difference in ACM or CVM rates was observed among the tertiles.

The C-indices of hs-cTnI and NT-proBNP for mortality at each year over the entire 7-year follow-up period are shown in Table S3. C-indices for ACM in the two biomarkers similarly decreased from 0.79 to 0.65 over the years, without significant difference in C-indices for ACM. The C-index for CVM at 4 years was significantly higher for hs-cTnI than for NT-proBNP, and those at 5–7 years were likely to be higher for hs-cTnI than for NT-proBNP. The cut-off values of hs-cTnI for ACM and CVM were 16 to 43 ng/L, within or slightly above the reference range, whereas those of NT-proBNP were approximately 3000 to 18,000 pg/mL, which corresponded to 20- to 100-fold the reference value.

### Prognostic values of hs-cTnI and NTproBNP for ACM and CVM

In the univariate Cox proportional regression analysis, both hs-cTnI and NT-proBNP levels, as continuous and categorical factors, were associated with the risk of ACM and CVM (Table 2). The independent risk factors for ACM were determined using multivariate Cox analysis in the main model comprising traditional risk factors (sex, dialysis vintage, history of cardiovascular diseases, and hypertension) and univariate risk factors (age, DM, GNRI, ERI, CRP level, and TG level; Table 2). When either of the two biomarkers was included in the main model, hs-cTnI and NT-proBNP as continuous variables were selected as independent risk factors [HR (95% confidence interval] for ACM [2.316 (1.382–3.882), *P* = 0.0014 and 1.517 (0.999–2.304), *P* = 0.0505, respectively; Table 3]. Independent risk factors for CVM were determined in the main model that included only univariate risk factors, considering a maximum of 40 CVM events. After adjusting for age, DM, and CRP levels, both hs-cTnI and NTproBNP were selected as independent risk factors for CVM [4.282 (2.275–8.061), *P* < 0.0001 and 1.791 (0.999–3.213), *P* = 0.0506, respectively; Table 5]. Similar results were found when either of the two biomarkers was used as a categorical variable (tertiles) and these were analyzed similarly as described above (Tables 4 and 6).

**Table 2:** Univariate Cox proportional regression analysis for all-cause and cardiovascular mortality.

Variable	All-cause mortality		Cardiovascular mortlity	
	HR (95% CI)	*P-*value	HR (95% CI)	*P-*value
Age, years	1.043 (1.023–1.063)	< 0.0001	1.032 (1.004–1.060)	0.0240
Sex, men vs. women	1.175 (0.749–1.843)	0.4815	1.026 (0.541–1.946)	0.9384
HD vintage, months	0.999 (0.997–1.001)	0.4304	0.998 (0.995–1.002)	0.2941
HT vs. non HT	0.811 (0.419–1.570)	0.5347	1.001 (0.356–2.812)	0.9992
DM vs. non DM	1.586 (1.018–2.471)	0.0417	2.401 (1.290–4.469)	0.0057
CVD vs. non CVD	1.451 (0.938–2.243)	0.0944	1.740 (0.933–3.246)	0.0816
Smoking status, yes vs. no	0.935 (0.527–1.660)	0.8181	0.829 (0.348–1.975)	0.6719
BMI, kg/m^2^	1.015 (0.961–1.072)	0.5943	1.017 (0.939–1.100)	0.6847
GNRI	0.948 (0.920–0.978)	0.0007	0.969 (0.925–1.015)	0.1797
ERI, μg/Hb (g/dL)/DW (kg)	3.472 (1.074–11.219)	0.0375	2.275 (0.387–13.364)	0.3629
Ca, mg/dL	0.976((0.709–1.345)	0.8837	1.197 (0.758–1.869)	0.4401
iP, mg/dL	0.929 (0.798–1.083)	0.3484	1.026 (0.827–1.274)	0.8146
CRP, mg/dL^a^	2.285 (1.515–3.445)	< 0.0001	2.452 (1.350–4.455)	0.0032
Ca x iP, (mg/dL)^2^	0.993 (0.977–1.010)	0.4188	1.005 (0.983–1.028)	0.6435
Kt/V	0.706 (0.360–1.387)	0.3128	0.987 (0.374–2.599)	0.9783
PCR, g/day	0.988 (0.973–1.003)	0.1093	0.996 (0.975–1.017)	0.7154
LDL-C, mg/dL	0.994 (0.986–1.003)	0.1735	1.003 (0.991–1.015)	0.6576
HDL-C, mg/dL	0.993 (0.977–1.010)	0.4490	0.996 (0.972–1.021)	0.7427
TG, mg/dL^a^	0.369 (0.137–0.997)	0.0492	0.273 (0.065–1.155)	0.0776
Adiponectin, + 1 mg/mL	1.013 (0.993–1.033)	0.2056	1.016 (0.989–1.044)	0.2529
hs-cTnI, ng/L^a^	3.486 (2.250–5.402)	< 0.0001	5.935 (3.413–10.320)	< 0.0001
hs-cTnI (category)^b^				
Low tertile	Ref		Ref	
Middle tertile	1.869 (1.007–3.470)	0.0474	3.545 (1.156–10.876)	0.0269
High tertile	3.487 (1.955–6.220)	< 0.0001	7.642 (2.640–22.127)	0.0002
NT-proBNP, pg/mL^a^	2.268 (1.559–3.301)	< 0.0001	1.043 (1.023–1.063)	0.0003
NT-proBNP (category)^c^			2.712 (1.579–4.659)	
Low tertile	Ref		Ref	
Middle tertile	2.292 (1.233–4.261)	0.0087	1.768 (0.685–4.563)	0.2387
High tertile	3.601 (1.983–6.539)	< 0.0001	4.207 (1.794–9.862)	0.0009

*HR* Hazard ratio, *HD* haemodialysis, *HT* hypertension, *DM* diabetes mellitus,* CVD* cardiovascular disease, *BMI* body mass index, *GNRI* Geriatric Nutritional Risk Index, *ERI* Erythropoietin Resistance Index, *CRP* C-reactive protein, *PCR* protein catabolic rate, *hs-cTnI* high-sensitivity cardiac troponin I, *NT-proBNP* N-terminal pro-brain natriuretic peptide.

^a^log10-transformed before analysis.

^b^The tertiles of hs-ctni were 14.6 and 31.1 ng/L.

^c^The tertiles of NT-proBNP were 2570 and 7570 pg/mL.

**Table 3:** Multivariable Cox proportional regression analysis for all-cause mortality in models including hs-cTnI and/or NT-proBNP as continuous variables.

Variable	Model 1		Model 2		Model 3		Model 3^a^	
	HR (95% CI)	*P*-value	HR (95% CI)	*P*-value	HR (95% CI)	*P*-value	HR (95% CI)	*P*-value
Age, years	1.034 (1.011–1.059)	0.0043	1.036 (1.012–1.060)	0.0031	1.033 (1.010–1.058)	0.0059	1.033 (1.012–1.053)	0.001
Sex men vs. women	1.144 (0.691–1.892)	0.6016	1.167 (0.712–1.911)	0.5405	1.169 (0.703–1.943)	0.547		
HD vintage, months	1.000 (0.998–1.002)	0.9301	1.000 (0.998–1.003)	0.8232	1.000 (0.998–1.002)	0.9606		
HT vs. non HT	0.669(0.332–1.347)	0.26	0.630(0.313–1.267)	0.1947	0.673(0.334–1.357)	0.2683		
DM vs. non DM	1.610 (0.955–2.714)	0.0741	1.663 (0.971–2.748)	0.0645	2.052 (0.938–4.492)	0.072		
CVD vs. non CVD	0.886 (0.549–1.429)	0.6198	0.963 (0.601–1.542)	0.8744	0.886 (0.548–1.430)	0.6192		
GNRI	0.987 (0.949–1.026)	0.5116	0.991 (0.953–1.031)	0.6587	0.987 (0.949–1.026)	0.5092		
ERI μg/Hb(g/dL)/DW(kg)	1.488 (0.380–5.823)	0.5678	1.598 (0.410–6.233)	0.4996	1.484 (0.377–5.837)	0.5722		
CRP, mg/dL^b^	1.516 (0.947–2.426)	0.0828	1.611 (1.010–2.570)	0.0452	1.477 (0.915–2.385)	0.1101	1.659 (1.084–2.538)	0.0198
TG, mg/dL^b^	0.755 (0.241–2.360)	0.629	0.643(0.210–1.966)	0.439	1.154 (0.711–1.874)	0.636		
hs-cTnI, ng/L^b^	2.316 (1.382–3.882)	0.0014			2.120 (1.167–3.851)	0.0136	2.595 (1.590–4.238)	0.0001
NT-proBNP, pg/mL^b^			1.517 (0.999–2.304)	0.0505	0.877 (0.423–1.819)	0.7236		

*HR* Hazard ratio, *HD* haemodialysis, *HT* hypertension, *DM* diabetes mellitus,* CVD* cardiovascular disease, *BMI* body mass index, *GNRI* Geriatric Nutritional Risk Index, *ERI* Erythropoietin Resistance Index, *CRP* C-reactive protein, *PCR* protein catabolic rate, *hs-cTnI* high-sensitivity cardiac troponin I, *NT-proBNP* N-terminal pro-brain natriuretic peptide.

^a^Multivariable stepwiae Cox regression analysis was performed in the indicated model, and the selected variables were described.

^b^log10-transformed before analysis.

^c^The tertiles of hs-cTnI were 14.6 and 31.1 ng/L.

^d^The tertiles of NT-proBNP were 2570 and 7570 pg/mL.

**Table 4:** Multivariable Cox proportional regression analysis for all-cause mortality in models including hs-cTnI and/or NT-proBNP as categorial variables.

Variable	Model 1		Model 2		Model 3		Model 3^a^	
	HR (95% CI)	*P*-value	HR (95% CI)	*P-*value	HR (95% CI)	*P-*value	HR (95% CI)	*P-*value
Age, years	1.036 (1.012–1.061)	0.0034	1.035 (1.011–1.059)	0.0046	1.034 (1.009–1.060)	0.007	1.033 (1.012–1.053)	0.0011
Sex men vs. women	1.182 (0.713–1.960)	0.5174	1.110 (0.677–1.820)	0.6792	1.182 (0.711–1.966)	0.5188		
HD vintage, months	1.000 (0.998–1.002)	0.532	1.000 (0.998–1.002)	0.923	1.000 (0.998–1.002)	0.9291		
HT vs. non HT	0.656 (0.325–1.323)	0.2387	0.597 (0.292–1.221)	0.1577	0.620 (0.301–1.279)	0.1959		
DM vs. non DM	1.653 (0.989–2.765)	0.0553	1.586 (1.018–2.471)	0.0727	1.550 (0.918–2.617)	0.101		
CVD vs. non CVD	0.876 (0.543–1.413)	0.5868	0.940 (0.589–1.502)	0.7965	0.867 (0.538–1.399)	0.5596		
GNRI	0.993 (0.955–1.032)	0.7253	0.992 (0.953–1.032)	0.6816	0.994 (0.956–1.035)	0.7787		
ERI μg/Hb (g/dL)/DW (kg)	1.686 (0.429–6.635)	0.4547	1.396 (0.354–5.495)	0.6335	1.448 (0.362–5.790)	0.6007		
CRP, mg/dL^b^	1.634 (1.028–2.599)	0.0378	1.773 (1.095–2.871)	0.0198	1.707 (1.045–2.788)	0.0328	1.818 (1.033–2.766)	0.0051
TG, mg/dL^b^	0.670 (0.220–2.046)	0.4822	0.601 (0.196–1.837)	0.3715	0.652 (0.212–2.010)	0.4566		
hs-cTnI (category)^c^								
Low tertile	Ref				Ref		Ref	
Middle tertile	1.263 (0.659–2.422)	0.4814			1.170 (0.608–2.252)	0.6383	1.344 (0.707–2.557)	0.3673
High tertile	2.111 (1.128–3.952)	0.0195			1.783 (0.898–3.540)	0.098	2.314 (1.260–4.249)	0.0068
NT-proBNP (category)^d^								
Low tertile			Ref		Ref			
Middle tertile			1.989 (1.047–3.779)	0.0357	1.838 (0.959–3.524)	0.0668		
High tertile			2.178 (1.157–4.098)	0.0159	1.687(0.841–3.383)	0.1402		

*HR* Hazard ratio, *HD* haemodialysis, *HT* hypertension, *DM* diabetes mellitus,* CVD* cardiovascular disease, *BMI* body mass index, *GNRI* Geriatric Nutritional Risk Index, *ERI* Erythropoietin Resistance Index, *CRP* C-reactive protein, *PCR* protein catabolic rate, *hs-cTnI* high-sensitivity cardiac troponin I, *NT-proBNP* N-terminal pro-brain natriuretic peptide.

^a^Multivariable stepwiae Cox regression analysis was performed in the indicated model, and the selected variables were described.

^b^log10-transformed before analysis.

^c^The tertiles of hs-cTnI were 14.6 and 31.1 ng/L.

^d^The tertiles of NT-proBNP were 2570 and 7570 pg/mL.

**Table 5:** Multivariable Cox proportional regression analysis for cardiovascular mortality in models including hs-cTnI and/or NT-proBNP as continuous variables .

Variable	Model 1		Model 2		Model 3		Model 3^a^	
	HR (95% CI)	*P*-value	HR (95% CI)	*P*-value	HR (95% CI)	*P*-value	HR (95% CI)	*P*-value
Age, years	1.026 (0.997–1.057)	0.0795	1.029 (1.000–1.059)	0.052	1.027 (0.997–1.058)	0.0785		
DM vs. non DM	1.867 (0.953–3.655)	0.0686	2.147 (1.110–4.156)	0.0233	1.885 (0.957–3.714)	0.0668		
CRP, mg/dL^b^	1.330 (0.712–2.487)	0.3709	1.706 (0.920–3.164)	0.09	1.344 (0.714–2.530)	0.3596		
hs-cTnI, ng/L^b^	4.282 (2.275–8.061)	< 0.0001			4.478 (2.078–9.649)	0.0001	5.935 (3.415–10.316)	< 0.0001
NT-proBNP, pg/mL^b^			1.791 (0.999–3.213)	0.0506	0.930 (0.457–1.889)	0.8400		

*HR* Hazard ratio, *DM* diabetes mellitus, *CRP* C-reactive protein, *hs-TnI* high-sensitivity cardiac troponin I, *NT-proBNP* N-terminal pro-brain natriuretic peptide.

^a^Multivariable stepwise Cox regression analysis was performed in the indicated model, and selected variables were described.

^b^log10-transformed before analysis.

^c^The tertiles of hs-cTnI were 14.6 and 31.1 ng/L.

^d^The tertiles of NT-proBNP were 2570 and 7570 pg/mL.

**Table 6:** Multivariable Cox proportional regression analysis for cardiovascular mortality in models including hs-cTnI and/or NT-proBNP as categorical groups.

Variable	Model 1		Model 2		Model 3		Model 3^a^	
	HR (95% CI)	*P*-value	HR (95% CI)	*P*-value	HR (95% CI)	*P*-value	HR (95% CI)	*P*-value
Age, years	1.026 (0.997–1.056)	0.0821	1.028 (0.999–1.058)	0.0606	1.024 (0.995–1.054)	0.1068		
DM vs. non DM	2.057 (1.069–3.959)	0.0309	2.154 (1.121–4.141)	0.0213	1.978 (1.020–3.834)	0.0434	1.897 (1.013–3.551)	0.0454
CRP, mg/dL^b^	1.640 (0.898–2.996)	0.1074	1.756 (0.943–3.270)	0.0756	1.601 (0.860–2.982)	0.1376		
hs-cTnI (category)^c^								
Low tertile	Ref				Ref		Ref	
Middle tertile	2.559 (0.812–8.066)	0.1087			2.420 (0.762–7.688)	0.134	3.284 (1.068–10.095)	0.0381
High tertile	4.822 (1.601–14.523)	0.0052			3.979 (1.212–13.058)	0.0227	6.695 (2.291–19.553)	0.0005
NT-proBNP (category)^d^								
Low tertile		0.532	Ref		Ref			
Middle tertile			1.481 (0.565–3.882)	0.4240	1.239 (0.466–3.291)	0.6675		
High tertile			2.570 (1.058–6.239)	0.0370	1.515(0.569–4.033)	0.4057		

*HR* Hazard ratio, *DM* diabetes mellitus, *CRP* C-reactive protein, *hs-TnI* high-sensitivity cardiac troponin I, *NT-proBNP* N-terminal pro-brain natriuretic peptide.

^a^Multivariable stepwise Cox regression analysis was performed in the indicated model, and selected variables were described.

^b^log10-transformed before analysis.

^c^The tertiles of hs-cTnI were 14.6 and 31.1 ng/L.

^d^The tertiles of NT-proBNP were 2570 and 7570 pg/mL.

When both biomarkers were added as continuous variables to the main model, hs-cTnI, but not NT-proBNP, was an independent risk factor for ACM [2.120 (1.167–3.851), *P* = 0.0136] and CVM [4.478 (2.078–9.649), *P* = 0.0001; Tables 3 and 5]. When the two biomarkers were added and analysed as categorical factors, neither hs-cTnI nor NT-proBNP levels were significantly associated with ACM, whereas the high tertile of hs-cTnI was an independent risk factor for CVM (Tables 4 and 6). Multivariable stepwise Cox proportional analysis revealed that hs-cTnI, as a categorical variable, but not NT-proBNP, was associated with ACM and CVM. The high tertile of hs-cTnI had a more than twofold and sixfold higher risk of ACM and CVM, respectively, and the middle tertile of hs-cTnI had a more than threefold higher risk of CVM than the low tertile (Tables 4 and 6). These results indicated that even low levels of hs-cTnI, such as levels in the upper half of the reference range (14.6–30.9 ng/L), may constitute risk factors for long-term mortality in HD patients, and that even HD patients with slightly elevated hs-cTnI levels warrant attention to modulate more appropriate long-term management.

Finally, to clarify whether the addition of hs-cTnI or NT-proBNP can reclassify patients for mortality more accurately compared with the main model that includes an alternative biomarker, the corresponding NRI and IDI at 1, 3, 5, and 7 years were calculated. The prognostic powers of the biomarker-added models for ACM and CVM are listed in Table 5. The addition of hs-cTnI to the main model including NT-proBNP significantly increased the NRI and IDI for ACM and CVM at almost all time points (Table 7). The addition of NT-proBNP to the main model including hs-cTnI significantly increased only the NRI for the CVM at 1 year (Table 7).

**Table 7:** Prognostic powers for all-cause and caridiovasucular mortality by adding hs-cTnI or NT-proBNP to a model that included an alternative biomarker by using NRI and IDI.

	NRI% (95%CI)	*P*-value	IDI% (95%CI)	*P*-value
All cause mortality (n = 84)				
hs-cTnI^a^				
1-year risk	53.3 (7.9–98.6)	0.021	6.8 (− 0.8–14.4)	0.080
3-year risk	34.8 (0.6–69)	0.046	3.8 (− 0.3–7.1)	0.076
5-year risk	42.78 (14.16–71.39)	0.003	3.2 (0.5–5.9)	0.014
7-year risk	30.69 (3.84–57.54)	0.02507	1.99 (0–3.99)	0.0507
NT-proBNP^b^				
1-year risk	43.3 (− 2.2–88.9)	0.062	1.8 (− 2.3–5.8)	0.396
3-year risk	23.4 (− 11–57.9)	0.182	1.0 (− 0.9–2.9)	0.308
5-year risk	4.74 (− 24.44–33.93)	0.750	0.21 (− 0.56–0.88)	0.594
7-year risk	0.6 (− 27.7–26.5)	0.967	0.02 (− 0.3–0.3)	0.903
Cardiovascular death (n = 40)				
hs-cTnI^c^				
1-year risk	64.4 (10.1–118.7)	0.0201	10.7 (− 0.6–21.9)	0.063
3-year risk	58.9 (16.6–101.2)	0.0006	9.5 (1.9–17.0)	0.014
5-year risk	70.51 (34.29–106.72)	0.0001	11.86 (4.77–18.96)	0.002
7-year risk	47.6 (14.0–81.2)	0.0055	8.6 (3.1–14.1)	0.002
NT-proBNP^d^				
1-year risk	67.3 (13.0–121.6)	0.015	5.6 (− 2.7–13.8)	0.187
3-year risk	33.7 (− 10.0–77.5)	0.131	2.0 (− 1.1–5.1)	0.206
5-year risk	− 15.29 (− 53.1–22.53)	0.428	0.02 (− 0.17–0.21)	0.840
7-year risk	17.8 (− 16.2–51.7)	0.305	0.2 (− 0.4–0.8)	0.532

*NRI* Net Reclassification Index, *IDI* Integrated Discrimination Index, *hs-cTnI* high-sensitivity cardiac troponin I, *NT-proBNP* N-terminal pro-brain natriuretic peptide.

^a^The basic model included history of hypertension, diabetes mellitus, and cardiovascular events, age, sex, haemodialysis vintage, ERI, GNRI, CRP, TG and NT-proBNP.

^b^The basic model included history of hypertension, diabetes mellitus, and cardiovascular events, age, sex, haemodialysis vintage, ERI, GNRI, CRP, TG and hs-cTnI.

^c^The basic model included age, history of diabetes mellitus, CRP, and NT-proBNP.

^d^The basic model included age, history of diabetes mellitus, CRP, and hs-cTnI.

## Discussion

In this study, we evaluated, for the first time, the prognostic potential of major cardiovascular markers, high-sensitivity cTnI (hs-cTnI), NT-proBNP, and adiponectin, for ACM and CVM in stable HD patients over a long follow-up period of 7 years. We found that hs-cTnI and NT-proBNP, but not adiponectin, were significant risk factors for ACM and CVM in univariate analysis using the Kaplan–Meier and Cox proportional hazards methods. The C-index of ACM for hs-cTnI and NT-proBNP decreased similarly over seven years, whereas the C-index of CVM at to 4–7 years was likely to be higher for hs-cTnI than for NT-proBNP. In multivariate Cox proportional regression analysis using clinical risk factors, including both hs-cTnI and NT-proBNP, the independent risk factors for ACM were hs-cTnI, age, and CRP, but not NT-proBNP, whereas the independent risk factors for CVM were hs-cTnI and DM, but not NT-proBNP. Furthermore, when hs-cTnI was added to the main model, including NT-proBNP in addition to age, CRP, and DM as independent prognostic risk factors, the NRI and IDI for ACM and CVM increased significantly throughout the entire period, leading to long-term improvement of the prognostic ability. Though the discriminatory ability of NT-proBNP was similarly evaluated, the NRI for the CVM increased significantly only in the first year. These results suggest that hs-cTnI is superior to NT-proBNP and adiponectin in predicting ACM and CVM over seven years in stable HD patients.

The correlation between biomarkers may have influenced the results of the analysis of independent risk factors. In this study, more than 99% of the measurements of hs-cTnI, NT-proBNP, and adiponectin were above the LOQ, and this confirmed the accuracy of the measurements. Based on this analysis, the main positive independent determinant of hs-cTnI was NT-proBNP, whereas the main positive determinants of NT-proBNP were DM, CRP, and hs-cTnI. In earlier reports on dialysis patients, the NT-proBNP level was positively associated with inflammation or CRP^9,25,26^. The association between NT-proBNP and several factors may partially explain why it was not selected as an independent risk factor for mortality after adjusting for age, DM and hs-cTnI levels. Adiponectin was not an independent risk factor for mortality in this study, probably because it was also independently and positively correlated with hs-cTnI levels, higher HDL-C levels, and female sex. Previous studies have also reported significant positive associations between adiponectin and cTnI^27^ and NT-proBNP^13,28^.

In the present study, the middle tertile (14.6–30.9 ng/L) and the high tertile (> 31.1 ng/L) were independently correlated with ACM and CVM, which demonstrated that even a mild increase in cTnI within the reference range was associated with poor prognosis. A large-scale prospective study of the general population revealed a cTnI concentration-dependent prediction of CVM at 13 years even in the lower half of the reference range, that is, ≤ 6 ng/L. This indicated that cTnI measurements within the reference range reflected the status of subclinical myocardial stress^29^. Myocardial troponin release is caused not only by necrosis and apoptosis of acute myocardial injury but also by chronic diseases, such as myocardial overload, myocardial wall strain, and myocardial ischemia^7^. Chronic myocardial stress leads to myocyte turnover, intracellular cTnI degradation, increased plasma membrane permeability, and plasma membrane bleb formation^30,31^. cTnI may be released from stressed viable cardiomyocytes via plasma membrane shedding of vesicular blebs containing cytoplasmic TnI^30^. In previous studies on dialysis patients, a cTnI cut-off value of 14–30 ng/L within the reference range had significant predictive value for ACM at 1–4 years^2,9,32^, with which our findings are aligned. The elevated cTnI levels in patients with renal insufficiency can be attributed to myocardial damage/stress and decreased clearance^3,7,9^. However, cTnI measurements were only slightly affected by reduced clearance, as clearance occurs in the liver and kidneys^33^. Approximately one-third of our participants had cTnI levels above the reference range. In contrast, NT-proBNP levels exceeded the reference range in almost all participants in our study because NT-proBNP mainly undergoes renal clearance^3^.

One of the most important findings of this study was that the baseline levels of hs-cTnI, but not NT-proBNP, were significant independent predictors of ACM and CVM at 7 years and had better prognostic discriminatory power for ACM and CVM than NT-proBNP throughout the 7-year follow-up period. cTnI and NT-proBNP had almost identical prognostic significance with 4-year follow-up^9^, and that cTnI had a slightly stronger predictive power for ACM than NT-proBNP, although both were significant predictors^2^. In these studies, most NT-proBNP measurements were above the LOQ, whereas more than 50% of the cTnI measurements were below the LOQ (17 and 30 ng/L)^2,9^. This partial imprecision of cTnI values may have resulted in failure to confirm the superiority of cTnI. In a study of HD patients using a highly sensitive assay with an LOQ of 3.8 ng/L, cTnI had a greater discriminatory power for mortality at 5 years compared to NT-proBNP^34^. In contrast, in a study of HD and peritoneal dialysis patients using a low-sensitivity cTnI assay with an LOQ of approximately 100 ng/L, NT-proBNP had a significantly greater predictive power for mortality at several years compared to cTnI^26,35^. Another reason may be differences in the intra-individual variability of the measurements. The reference change values of high-sensitivity cTnI were approximately half of those of NT-proBNP^36–38^. Accordingly, high-sensitivity cTnI may be more likely than NT-proBNP to reflect fundamental and persistent cardiac conditions in a single measurement and may be more strongly associated with long-term prognosis than NT-proBNP. Additional explanation may be the differences in dialysis modalities, considering that in studies on patients on peritoneal dialysis, NT-proBNP was reported to be a stronger predictor of mortality than high-sensitivity troponin^25,36^.

Few studies have examined the long-term prognostic value of biomarkers over 5 years in HD patients. In an earlier study of Japanese HD patients, the AUCs of NT-proBNP for both ACM and CVM gradually decreased at 1, 3, and 5 years^39^, which was similar to our findings for NT-proBNP throughout the 7-year study period. In a report using non-high-sensitivity cTnI, the discrimination ability for sudden cardiac death was significantly greater with NT-proBNP than with cTnI at both 3 and 5 years^26^. In contrast, in the present study, hs-cTnI was superior to NT-proBNP in the discrimination ability for ACM and CVM from 1 to 7 years of age, with CVM being much greater. The difference between the earlier report and ours may be attributed to the difference in the sensitivity of the cTnI assay.

As shown in Table 2, ERI showed a univariate predictor of ACM but not CVM, while preexisting CVD was a univariate weak predictor of clinical outcomes in contrast to hs-cTnI as the powerful predictor. These unique associations of ERI and preexisting CVD with the outcomes can be explained as follows: ERI showed a univariate positive association with age (β = 0.236, P < 0.001) and negative ones with BMI (β = − 0.333, P < 0.001), GNRI (β = − 0.331, P < 0.001) and TG levels (β = − 0.278, P < 0.001). ERI was associated with 3 univariate predictors of ACM, age, GNRI, and TG, and with one univariate predictor of CVM: only age. This difference may cause a stronger univariate association of ERI with ACM than CVM (Table 2). Next, preexisting CVD showed weak univariate positive associations with risk factors of the outcomes: DM (β = 0.265, P < 0.001), CRP (β = 0.162, P < 0.05), hs-cTnI (β = 0.230, P < 0.001) and NT-proBNP (β = 0.160, P < 0.05). CVD patients were considered to take preventive treatments for recurrence before the participation, which may lead to reduced levels of CRP, hs-cTnI, and NT-proBNP. Consequently, associations between these risk factors and preexisting CVD may have weakened. Furthermore, no significant difference in age was found between CVD and non-CVD patients (68.7 ± 10.9 vs 65.9 ± 13.2 years, P = 0.12). These can explain why associations of preexisting CVD with ACM and CVM were much weaker than those of hs-cTnI.

Finally, this study found weak positive associations of eGFR with cTnI, NT-proBNP, and age, contrary to general expectations. In this study, eGFR values were calculated from the Japanese equation using Cr levels^16^. Consequently, they depended mainly on Cr levels, where the two factors had a strong inverse relationship (β = − 0.823, P < 0.001). Since Cr levels were associated negatively with age (β = − 0.513, P < 0.001) and adiponectin (β = − 0.287, P < 0.001) and positively with GNRI (β = 0.413, P < 0.001), eGFR was resultantly associated positively with age and adiponectin and negatively with GNRI as shown in the Result section. Furthermore, considering an earlier report that 69% of HD patients had residual renal function (RRF) loss, defined as a urine volume below 200 mL/day, 20 months after HD initiation^40^, most of our participants may have presented with RRF loss, because of having the median HD vintage of 86 months. Taking these into account, eGFR only marginally reflected renal clearance and showed no negative association with cTnI or NT-proBNP. Furthermore, cTnI and NT-proBNP were associated positively with age and adiponectin and negatively with GNRI. These correlations were similar to those found for eGFR, which may have led to the weak positive associations of eGFR with cTnI and NT-proBNP.

This study has several limitations. First, the results of this single-centre small-scale clinical study need to be validated in a large-scale, long-term prospective multicentre study that quantifies the hs-cTnI. Second, the methods for measuring hs-cTnI, NT-proBNP, and adiponectin levels have not been standardised or harmonised, and the cut-off values cannot yet be strictly applied to uraemic patients. Third, this study evaluated the prognostic performance of a single measurement but not the average value of multiple measurements. Some studies reported no difference in the prognostic value between single and multiple measurements^41^, whereas others reported that multiple measurements were better^36,37^. Fourth, though cTnI may be adsorbed onto dialyser membranes^42^, this study did not compare the pre- and post-dialysis levels; therefore, further investigation on this aspect is needed.

In conclusion, after adjusting for clinical risk factors, we demonstrated that hs-cTnI was superior to NT-proBNP and adiponectin for predicting and discriminating ACM and CVM over 7 years in patients who were undergoing HD, suggesting the significance of baseline hs-cTnI measurements in long-term management of patients who require HD. To validate these findings, a large-scale, long-term, prospective, multicentre study using hs-cTnI assay is required.

## Supplementary Information

Supplementary Tables.

## Data Availability

The data underlying this article will be shared upon reasonable request from the corresponding author (H.K.: hkimura@u-fukui.ac.jp).
