# Author Correction: Superiority of high sensitivity cardiac troponin I over NT‑proBNP and adiponectin for 7‑year mortality in stable patients receiving haemodialysis

**DOI:** 10.1038/s41598-025-89201-y

**Published:** 2025-02-12

**Authors:** Nanami Iwamura, Shuhei Kidoguchi, Nanae Asahi, Izumi Takeda, Kohei Matsuta, Kyoko Miyagi, Masayuki Iwano, Ryoichi Miyazaki, Hideki Kimura

**Affiliations:** 1 Department of Clinical Laboratory, University of Fukui Hospital, 23-3 Matsuoka-Shimoaizuki, Eiheiji, Yoshida, Fukui, 910-1193 Japan; 2 Department of Internal Medicine, Fujita Memorial Hospital, Fukui, Japan; 3 Division of Nephrology, Department of General Medicine, School of Medicine, University of Fukui, Fukui, Japan

Correction to: *Scientific Reports* 10.1038/s41598-024-62491-4, published online 20 May 2024

In the original version of this article, Tables 2 and 3 contained errors in four instances of the statistical results for ‘TG’ as a variable.

The correct and incorrect data appear below.

Incorrect Table 2:

**Table:** 

Variable	All-cause mortality		Cardiovascular Mortality	
	HR (95% CI)	*P-value*	HR (95% CI)	*P-value*
TG, mg/dL^a^	0.369 (0.137–0.997)	0.532	0.273 (0.065–1.155)	0.0776

Correct Table 2:

**Table:** 

Variable	All-cause mortality		Cardiovascular Mortality	
	HR (95% CI)	*P-value*	HR (95% CI)	*P-value*
TG, mg/dL^a^	0.369 (0.137–0.997)	0.0492	0.273 (0.065–1.155)	0.0776

Incorrect Table 3:

**Table:** 

Variable	Model 1		Model 2		Model 3	
	HR (95% CI)	*P-value*	HR (95% CI)	*P-value*	HR (95% CI)	*P-value*
TG, mg/dL^b^	0.755 (0.241–2.360)	0.0031	0.643(0.210–1.966)	0.0031	1.154 (0.711–1.874)	0.0031

Correct Table 3:

**Table:** 

Variable	Model 1		Model 2		Model 3	
	HR (95% CI)	*P-value*	HR (95% CI)	*P-value*	HR (95% CI)	*P-value*
TG, mg/dL^b^	0.755 (0.241–2.360)	0.629	0.643(0.210–1.966)	0.439	1.154 (0.711–1.874)	0.636

The original Article has been corrected.

