# Supplementary material for: Superiority of high sensitivity cardiac troponin I over NT-proBNP and adiponectin for 7-year mortality in stable patients receiving haemodialysis
Source: Sci Rep. 2024 May 20;14:11488. doi: 10.1038/s41598-024-62491-4 (PMC11106234; doi:10.1038/s41598-024-62491-4)
Supplement: Supplementary file 1 — Supplementary Tables. [file 41598_2024_62491_MOESM1_ESM.pdf]

Supplementary information

Superiority of high sensitivity cardiac troponin I over NT-proBNP and adiponectin for 7-year mortality in stable patients receiving haemodialysis

Nanami Iwamura[1], Shuhei Kidoguchi[1], Nanae Asahi[1], Izumi Takeda[1], Kohei Matsuta[1], Kyoko Miyagi[2], Masayuki Iwano[3], Ryoichi Miyazaki[2], Hideki Kimura[1*]

1 Department of Clinical Laboratory, University of Fukui Hospital, Fukui, Japan

2 Department of Internal Medicine, Fujita Memorial Hospital, Fukui, Japan

3 Division of Nephrology, Department of General Medicine, School of Medicine, University of Fukui, Fukui, Japan

*Name and address of the author to whom communications regarding the manuscript should be directed;

Hideki Kimura, MD, PhD

Department of Clinical Laboratory, University of Fukui Hospital, Fukui, Japan 23-3 Matsuoka-shimoaizuki, Eiheiji, Yoshida, Fukui 910-1193, Japan

Tel. No. : +81-776-61-3111 (EXT. 2412)

Fax. No. : +81-776-61-8120

E-mail address: hkimura@u-fukui.ac.jp

Table S1A Univariate and multivariate linear regression analyses for identifying clinical factors that affect serum hs-cTnI levels

	hs-cTnI[a]								
	Univariate linear[b]		Multivariate linear[b]					Stepwise[c]	
	β	*P*-value	β	95% CI			*P*-value	β	*P*-value
Age, years	0.226	<0.001	0.086	-0.041	to	0.213	ns	—	
Men vs.Women (1 vs. 0)	0.036	ns	—				—	—	
DM vs. non DM (1 vs. 0)	0.153	<0.05	-0.004	-0.127	to	0.118	ns	—	
CVD vs. non CVD (1 vs.0)	0.230	<0.001	0.115	-0.001	to	0.232	ns	0.145	<0.05
BMI, kg/m^2	0.037	ns	—					—	
GNRI	-0.156	<0.05	0.064	-0.074	to	0.201	ns	—	
ERI, mg/Hb(g/dL)/DW(kg)	0.143	<0.05	-0.028	-0.151	to	0.094	ns	—	
CRP, mg/dL[a]	0.261	<0.001	0.111	-0.011	to	0.234	ns	—	
eGFR, mL/min/1.73m^2 [c]	0.155	<0.05	0.030	-0.094	to	0.153	ns	—	
Kt/V	-0.007	ns	—				—	—	
PCR, g/day	0.055	ns	—				—	—	
LDL-C, mg/dL	-0.068	ns	—				—	—	
HDL-C, mg/dL	-0.046	ns	—				—	—	
TG, mg/dL[a]	-0.159	<0.05	-0.065	-0.183	to	0.053	ns	—	
Adiponectin, mg/mL	0.212	<0.01	0.063	-0.063	to	0.190	ns	—	
logNT-proBNP, pg/mL[a]	0.557	<0.001	0.482	0.356	to	0.609	<0.001	0.534	<0.001

Table S1B Univariate and multivariate linear regression analyses for identifying clinical factors that affect serum NT-proBNP levels

	NT-proBNP[a]								
	Univariate linear[b]		Multivariate linear[b]					Stepwise[c]	
	β	*P*-value	β	95% CI			*P*-value	β	*P*-value
Age, years	0.237	<0.001	0.020	-0.105	to	0.144	ns	—	
Men vs.Women (1 vs. 0)	-0.108	ns	—				—	—	
DM vs. non DM (1 vs. 0)	0.215	<0.01	0.205	0.091	to	0.320	<0.001	0.195	<0.001
CVD vs. non CVD (1 vs.0)	0.160	<0.05	-0.010	-0.121	to	0.102	ns	—	
BMI, kg/m^2	-0.187	<0.01	-0.169	-0.305	to	-0.033	<0.05	-0.269	<0.001
GNRI	-0.253	<0.001	-0.027	-0.170	to	0.116	ns	—	
ERI, mg/Hb(g/dL)/DW(kg)	0.244	<0.001	0.073	-0.041	to	0.187	ns	—	
CRP, mg/dL[a]	0.272	<0.001	0.160	0.043	to	0.277	<0.01	0.151	<0.01
eGFR, mL/min/1.73m^2 [c]	0.137	<0.05	-0.112	-0.232	to	0.008	ns	—	
Kt/V	0.110	ns	—				—	—	
PCR, g/day	-0.170	<0.05	-0.118	-0.247	to	0.011	ns	—	
LDL-C, mg/dL	-0.121	ns	—				—	—	
HDL-C, mg/dL	-0.019	ns	—				—	—	
TG, mg/dL[a]	-0.127	ns	—				—	—	
Adiponectin, mg/mL	0.278	<0.001	0.080	-0.040	to	0.201	ns	—	
hs-cTnI, ng/L[a]	0.557	<0.001	0.478	0.361	to	0.595	<0.001	0.497	<0.001

Table S1C Univariate and multivariate linear regression analyses for identifying clinical factors that affect serum adiponectin levels

	Adiponectin								
	Univariate linear[b]		Multivariate linear[b]					Stepwise[b]	
	β	*P*-value	β	95% CI			*P*-value	β	*P*-value
Age, years	0.211	<0.01	0.039	-0.085	to	0.163	ns	—	
Men vs.Women (1 vs. 0)	-0.293	<0.001	-0.191	-0.336	to	-0.047	<0.05	-0.237	<0.001
DM vs. non DM (1 vs. 0)	0.014	ns	—				—	—	
CVD vs. non CVD (1 vs.0)	0.087	ns	—				—	—	
BMI, kg/m^2	-0.369	<0.001	-0.166	-0.311	to	-0.021	<0.05	-0.229	<0.001
GNRI	-0.235	<0.001	—				—	—	
ERI, mg/Hb(g/dL)/DW(kg)	0.292	<0.001	0.097	-0.024	to	0.217	ns	—	
CRP, mg/dL[a]	-0.118	ns	-0.090	-0.213	to	0.032	ns	—	
eGFR, mL/min/1.73m^2 [c]	0.115	ns	—				—	—	
Kt/V	0.216	<0.01	-0.024	-0.165	to	0.117	ns	—	
PCR, g/day	-0.260	<0.001	-0.027	-0.171	to	0.116	ns	—	
LDL-C, mg/dL	-0.001	ns	—				—	—	
HDL-C, mg/dL	0.431	<0.001	0.285	0.162	to	0.408	<0.001	0.285	<0.001
TG, mg/dL[a]	-0.302	<0.001	-0.118	-0.245	to	0.009	ns	-0.144	<0.05
hs-cTnI, ng/L[a]	0.212	<0.01	0.159	0.022	to	0.296	<0.05	0.223	<0.001
NT-proBNP, pg/mL[a]	0.278	<0.001	0.120	-0.018	to	0.258	ns	—	

Abbreviations: Diabetes mellitus, DM; cardiovascular disease, CVD; body mass index, BMI; Geriatric Nutritional Risk Index, GNRI; Erythropoietin Resistance Index, ERI ; CRP, C-reactive protein; protein catabolic rate, PCR; high-sensitivity cardiac troponin I, hs-cTnI; N-terminal pro-brain natriuretic peptide, NT-proBNP

[a] log10-transformed before analysis

[b] β indicates standard partial regression coefficient.

[c] natural log-transformed before analysis

Table S2A. All causes of mortality during the 7-year follow-up period

All-cause mortality	84	
Cardiovascular death	40	(47.6 %)
Infection and sepsis	22	(26.2 %)
Malignancy	5	(6.0 %)
Wasting/cachexia	6	(7.1 %)
Hepatic failure/ intestinal perforation	5	(6.0 %)
others	6	(7.1 %)

Table S2B. Causes of cardiovascular mortality during the 7-year follow-up period

Causes of cardiovascular mortality	40	
Sudden death	17	(42.5 %)
Cardiac failure	12	(30.0 %)
Myocardial infarction	3	(7.5 %)
Stroke	6	(15.0 %)
Peripheral artery disease	1	(2.5 %)
Pulmonary hypertension	1	(2.5 %)

Table S3. C-statistics of the association of hs-cTnI and NT-proBNP with all-cause and cardiovascular mortality rates

All-cause mortality (n=84)										
hs-cTnI					NT-proBNP					
Years	AUC[a]	Cutoff (pg/mL)	Sensitivity	Specificity	Years	AUC[a]	Cutoff (pg/mL)	Sensitivity	Specificity	*P* -value[a]
1	0.794	34.9	0.750	0.776	1	0.795	4970	0.900	0.597	0.98
2	0.799	33.9	0.750	0.761	2	0.789	4970	0.875	0.604	0.83
3	0.719	33.9	0.590	0.769	3	0.747	2970	0.923	0.456	0.51
4	0.695	17.7	0.788	0.538	4	0.696	2530	0.923	0.391	0.97
5	0.714	17.7	0.794	0.563	5	0.686	2970	0.825	0.475	0.52
6	0.702	17.7	0.753	0.576	6	0.661	2970	0.779	0.479	0.31
7	0.679	16.7	0.762	0.526	7	0.650	2970	0.774	0.489	0.45
Cardiovascular mortality (n=40)										
hs-cTnI					NT-proBNP					
Years	AUC[a]	Cutoff (pg/mL)	Sensitivity	Specificity	Years	AUC[a]	Cutoff (pg/mL)	Sensitivity	Specificity	*P* -value[a]
1	0.783	34.9	0.769	0.760	1	0.803	18300	0.692	0.865	0.879
2	0.796	33.9	0.765	0.745	2	0.788	18300	0.647	0.873	0.884
3	0.794	33.9	0.727	0.754	3	0.770	9110	0.682	0.779	0.567
4	0.786	43.8	0.586	0.849	4	0.703	9110	0.586	0.781	0.0455
5	0.773	43.8	0.581	0.853	5	0.698	8640	0.581	0.763	0.0664
6	0.748	43.8	0.543	0.855	6	0.680	8640	0.571	0.769	0.156
7	0.723	16.9	0.850	0.481	7	0.651	8640	0.525	0.768	0.119

Abbreviations: area under the curve, AUC; high-sensitivity cardiac troponin I, hs-cTnI; N-terminal pro-brain natriuretic peptide, NT-proBNP

a: Statistical differences between AUCs of hs-cTnI and NT-proBNP in the same years of follow-up were determined.
